# Streamlining the institutional review board process in pragmatic randomized clinical trials: challenges and lessons learned from the Aspirin Dosing: A Patient-centric Trial Assessing Benefits and Long-Term Effectiveness (ADAPTABLE) trial

**DOI:** 10.1186/s13063-021-05026-w

**Published:** 2021-01-25

**Authors:** Guillaume Marquis-Gravel, Holly Robertson, W. Schuyler Jones, Danielle Riley, Daniel E. Ford, David Crenshaw, Yvonne A. Joosten, Lindsey Rudov, Adrian F. Hernandez, Rachel Hess

**Affiliations:** 1 Duke Clinical Research Institute, 200 Morris St, Durham, NC 27701 USA; 2 Duke University Medical Center, 2301 Erwin Road, Durham, NC 27710 USA; 3 University of Iowa College of Public Health, 145 N Riverside Dr, Iowa City, IA 52242 USA; 4 Institute for Clinical and Translational Research, Johns Hopkins School of Medicine, 750 E. Pratt Street, Baltimore, MD 21202 USA; 5 Institute for Medicine and Public Health, Vanderbilt University Medical Center, 2525 West End Avenue, Suite 1200, Nashville, TN 37203 USA; 6 Louisiana Public Health Institute, 1515 Poydras St #1200, New Orleans, LA 70112 USA; 7 Departments of Population Health Sciences and Internal Medicine, University of Utah School of Medicine, 295 Chipeta Way Williams Building Room 1N492, Salt Lake City, UT 84108 USA

**Keywords:** Institutional review boards, Ethics, Pragmatic trials, Informed consent, Aspirin, Cardiology

## Abstract

**Background:**

New considerations during the ethical review processes may emerge from innovative, yet unfamiliar operational methods enabled in pragmatic randomized controlled trials (RCT), potentially making institutional review board (IRB) evaluation more complex. In this manuscript, key components of the pragmatic “Aspirin Dosing: A Patient-Centric Trial Assessing Benefits and Long-term Effectiveness (ADAPTABLE)” randomized trial that required a reappraisal of the IRB submission, review, and approval processes are discussed.

**Main text:**

ADAPTABLE is a pragmatic, multicenter, open-label RCT evaluating the comparative effectiveness of two doses of aspirin widely used for secondary prevention (81 mg and 325 mg) in 15,000 patients with an established history of atherosclerotic cardiovascular disease. The electronic informed consent form is completed online by the participants at the time of enrollment, and endpoint ascertainment is conducted through queries of electronic health records.

IRB challenges encountered regarding centralized IRB evaluation, electronic informed consent, patient engagement, and risk determination in ADAPTABLE are described in this manuscript. The experience of ADAPTABLE encapsulates how pragmatic protocol components intended to facilitate the study conduct have been tempered by unexpected, yet justified concerns raised by local IRBs. How the lessons learned can be applied to future similar pragmatic trials is delineated.

**Conclusion:**

Development of engaging communication channels between IRB and study personnel in pragmatic randomized trials as early as at the time of protocol design allows to reduce issues with IRB approval. Integrations of the lessons learned in ADAPTABLE regarding the IRB process for centralized IRBs, informed consent, patient engagement, and risk determination can be emulated and will be instrumental in future pragmatic studies.

**Supplementary Information:**

The online version contains supplementary material available at 10.1186/s13063-021-05026-w.

## Background

High-quality comparative-effectiveness research is made complex by the growing burden of administrative requirements, frequent operational inefficiencies, and large sample sizes required to detect treatment effects in heterogeneous patient populations [1–4]. As comparative effectiveness research grows, strategies to streamline components of contemporary randomized controlled trials (RCT) will be needed to deliver simpler, faster, and more impactful research [5]. The integration of innovative approaches and of new technologies promises to fill this gap [6–10].

Ethical and regulatory reviews from institutional review boards (IRB) (or local equivalents) represent key requirements for all clinical research studies. IRBs are institutional committees with the role of ensuring the welfare, safety, privacy, and autonomy of human research participants, in accordance with Good Clinical Practice Guidelines and local standards [11]. Their role is to approve, disapprove, and/or ask modifications to research protocols, informed consent forms, protocol amendments, and any other study material for research involving humans. They are required to be informed by the investigators of any new event in the trial that could impact the participants’ safety. Its members are formally nominated and usually include at least one member whose primary concerns are not in the scientific area. Each project is reviewed before it is launched, as well as periodically afterwards, to make sure the rights of the subjects are respected. The results of the review process and its timeliness often vary across sites. As new methods are used to improve the participant experience or to conduct pragmatic clinical trials more integrated with clinical practice, new considerations may emerge from innovative, yet unfamiliar operational methods during the IRB review. These considerations can potentially make an IRB’s evaluation of RCTs more complex and extend the time to activation of individual study sites.

In this manuscript, we will summarize key lessons learned and progress brought about by the “Aspirin Dosing: A Patient-Centric Trial Assessing Benefits and Long-term Effectiveness (ADAPTABLE)” trial that required a reappraisal of the IRB submission, review, and approval processes. Challenges encountered related to the innovative and pragmatic components of ADAPTABLE (electronic informed consent, patient engagement, and risk determination) will be discussed, as well as their proposed solutions.

## Main text

### Overview of ADAPTABLE

ADAPTABLE, a pragmatic, multicenter, open-label, randomized controlled trial, is the first interventional study conducted broadly across PCORnet®, the National Patient-Centered Clinical Research Network, funded by the Patient-Centered Outcomes Research Institute (PCORI) [9, 12]. In brief, the primary objective is to evaluate the comparative effectiveness of two doses of aspirin widely used for secondary prevention (81 mg and 325 mg) in patients with an established history of atherosclerotic cardiovascular disease [13]. PCORnet®, a network of Clinical Research Networks (CRN) (networks of multiple independent healthcare systems), health plans research networks (HPRN), and patient-powered research networks (PPRN), was created to conduct patient-centered research, streamline study processes, and develop efficiencies that could be iteratively improved and replicated in a sustainable fashion. A key secondary aim of ADAPTABLE is to develop and evaluate PCORnet’s platform to refine the conduct of future trials undertaken within the network.

**Fig. 1:**
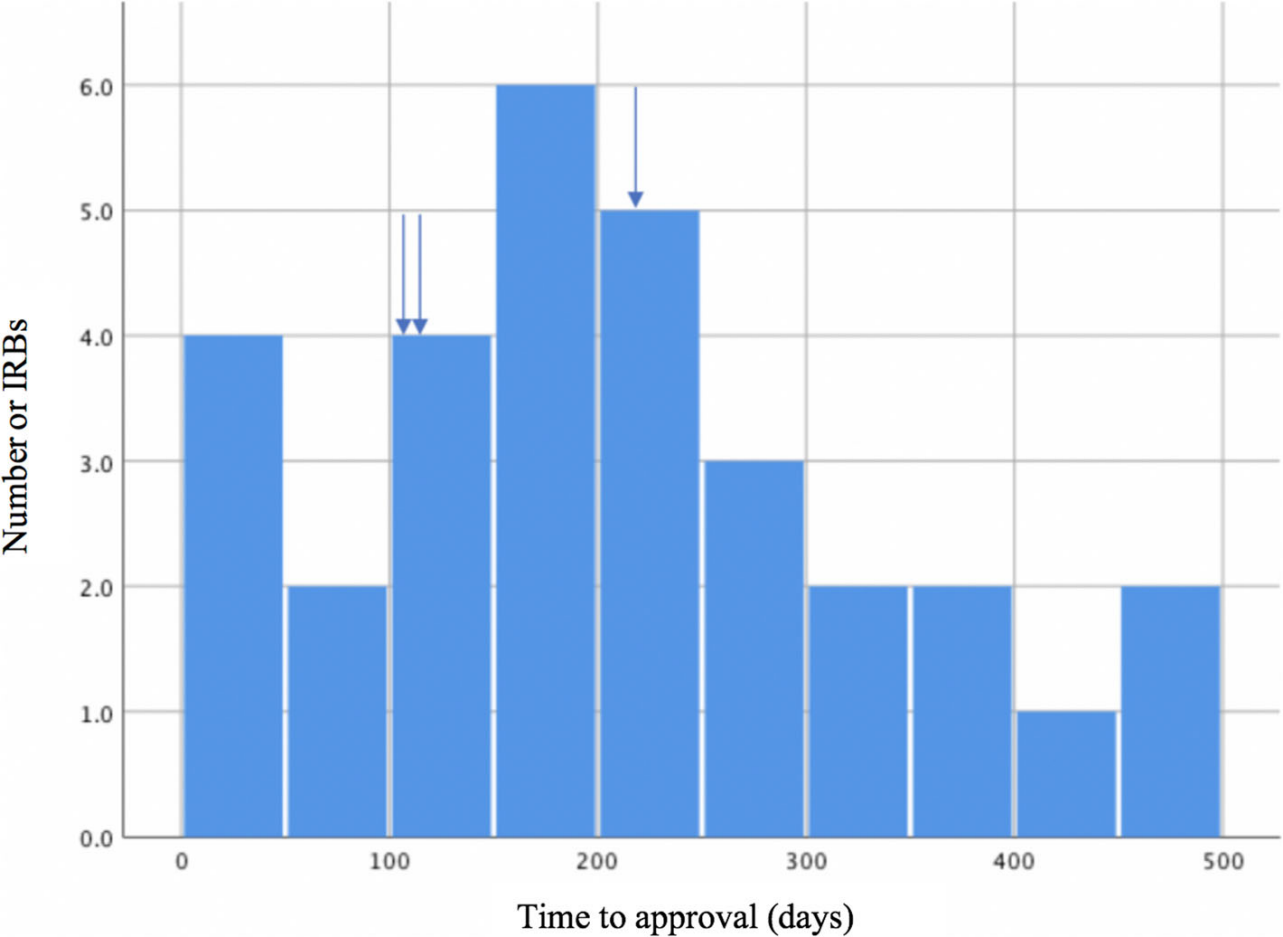
Distribution of time from shipment of regulatory package and primary IRB approval in ADAPTABLE study sites. *Arrows indicate multi-site approvals in three Clinical Data Networks*

As a pragmatic study, the design prioritizes integration of study processes seamlessly within usual clinical care and participants’ lives. The electronic consent is completed online by potential participants at the time of enrollment. No in-person study visit is required for the trial. Endpoint ascertainment and safety monitoring are facilitated by a common data model composed of cleaned, curated, local electronic health record (EHR) data. This endpoint ascertainment method is complemented by linkage of claims data, as well as collection of patient-reported outcomes through an online portal. As some innovations in specific components of the study design of ADAPTABLE represented novel concepts for most investigators and IRBs involved, unique challenges were encountered. The median time from distribution of the regulatory package for primary institutional review and approval across study sites was of 199 days (inter-quartile range 111–253 days), ranging from 31 to 474 days. In three CRNs, the IRB approval was centralized for multiple study sites and approval times were respectively 111, 125, and 230 days (Fig. 1).

### Centralized vs. individual IRB evaluations

As a pragmatic trial, ADAPTABLE study leadership aimed for IRB simplicity. The study protocol, informed consent form (ICF), and recruitment material were initially expected to be reviewed by a single centralized IRB to eliminate redundant reviews of the same material across multiple sites, to create efficiencies, and to hasten site activation. Due to heterogeneous IRB governance policies across participating ADAPTABLE sites, and to the lack of fully integrated reliance agreements, many CRNs and sites were not able to fully cede IRB regulatory oversight to a centralized body. Reliance agreements represent deferral of IRB oversight from one local IRB to another, unrelated local IRB from another study site. The IRB cedes the evaluation process of human research conducted on its study site to the IRB from another site. This way, a single IRB can approve a study for multiple study sites, as agreed upon in the reliance agreement. Most of the sites participated either in a reliance process in ADAPTABLE (a single IRB within the CRN approved for all participating sites within this CRN) or in a tiered-reliance process, in which a single IRB within the network approved the study, followed by local administrative approval in each participating site. Only a minority of CRNs utilized independent institutional reviews by the local IRBs for each participating site within their network.

Many unforeseen challenges unique to local IRBs were encountered during the local review processes, which might have been avoided with a single, centralized IRB. Regulatory liability was raised as a primary concern limiting the acceptability of the utilization of a centralized IRB in ADAPTABLE. In particular, some institutions did not want to take the responsibility of regulatory noncompliance resulting from a potential overlook by an external IRB and thus preferred their local IRB to review the protocol, despite the fact that the Office of Human Research Protections (OHRP) policy decouples institutional responsibilities from ethical reviews accountability. In fact, the institution in which the research is conducted cannot be held accountable for noncompliance to certain ethical regulatory requirements if an external IRB oversees the project [14]. In that regards, the Clinical Trials Transformation Initiative (CTTI) developed a tool to facilitate the acceptability and application of central IRBs in multi-center trials [15]. In future trials, dissemination of this policy and of this tool can be leveraged in pushing for the use of a single, central IRB across participating sites [15], addressing this challenge encountered in ADAPTABLE.

Because we were unable to use a single central IRB, we encountered additional challenges due to the varying levels of tolerance and experience for the novel concepts such as e-consent, remote enrollment, and siteless follow-up. Local IRBs address their concerns with the trial by requesting local amendments to the protocol or to study material, which were necessary at the majority of ADAPTABLE’s study sites. While challenges still occurred in centers that used central IRBs, which did not necessarily have more experience with our trial’s methods, addressing concerns and requests from one centralized IRB is operationally more efficient than addressing requests on a case-by-case basis from a multitude of local IRBs. In ADAPTABLE, IRB variation across sites was identified as a challenge at the time of review and start-up by local study teams. These challenges were addressed in a piecemeal fashion by keeping channels of communication opened between the local investigators, their IRBs, and the study coordinating center. However, the sum of these processes was time- and resource-consuming for all the stakeholders involved, despite the distribution of a consent template highlighting all the regulatory requirements to the IRBs involved (Additional file 1).

In the future, a continued movement towards acceptance of the concept of a central IRB with reliance agreements is expected to facilitate study start-up, to reduce costs, and to diminish inefficiencies at the local and central levels, without compromising participant safety. The support from the Food & Drug Administration (FDA) and the National Institutes of Health (NIH) in regard to single-center IRBs for multi-center human trials can be put forward at the time of the first contact with study sites to provide credibility to this approach from the get-go [16, 17]. The mandatory requirements for single IRB for all the multi-site research conducted with human participants funded by the NIH will help this practice gain momentum [17]. Ultimately, IRB harmonization at a national level will be enabled by education of IRB leaders, experience with centralized IRB, and consideration of a multi-stakeholder perspective.

### Electronic informed consent

In ADAPTABLE, the informed consent process was conducted electronically through the study’s online portal. Known as “e-consent”, this procedure has only recently gained support from the FDA [18]. The ADAPTABLE portal guides’ potential participants through a multi-step consent process, including (1) short video describing study expectations, (2) simplified text describing key aspects of consent, (3) brief study knowledge review questions to answer before signing the consent form, (4) key exclusion criteria, and (5) e-consent signature and randomization. This information is also available in a text version for participants. The information provided in the video is summarized in Table 1.

**Table 1:** Information provided in laypeople language in the online video consent module of the ADAPTABLE trial

∙ Background information on the use of aspirin in cardiovascular diseases; ∙ Description of the clinical equipoise regarding the optimal dose in secondary prevention; ∙ Goals of the ADAPTABLE trial; ∙ Randomization process; ∙ Study outcomes; ∙ Description of participants flow during the study; ∙ Study duration; ∙ Security of personal information; ∙ Side effects of aspirin; ∙ Absence of direct benefits to participate in the trial; ∙ Contact information of the study team.	

**Fig. 2:**
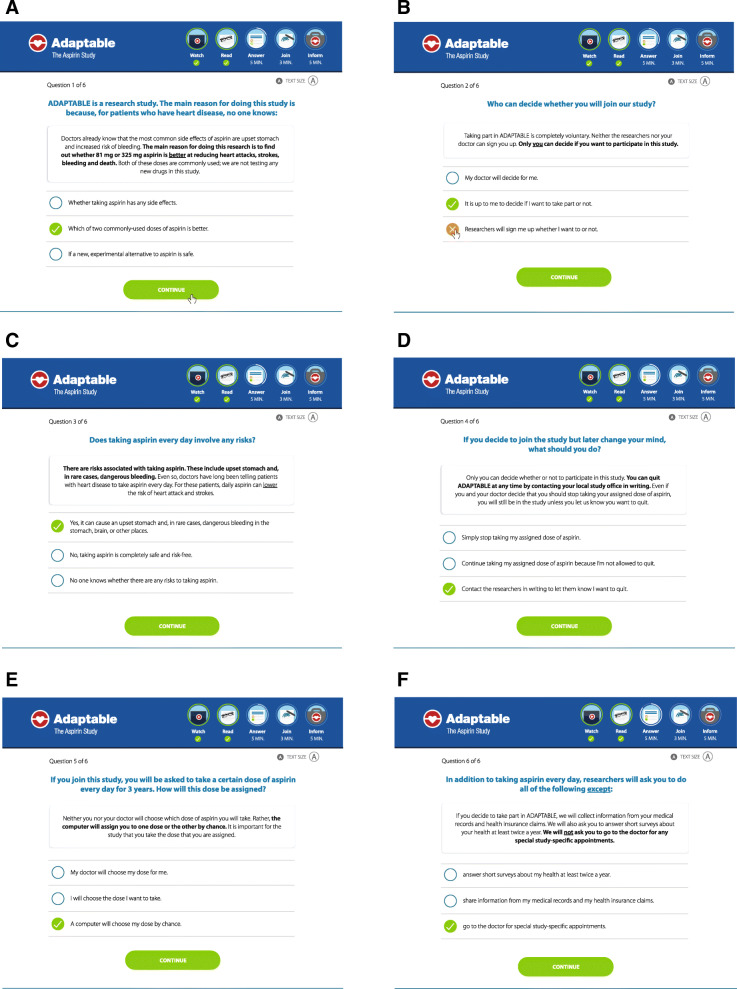
Informed consent study comprehension tool integrated to the e-consent online portal, developed by Duke’s program for empirical bioethics through iterative cognitive interviews with patients. After watching a video summarizing the trial, participants answer 6 questions to verify their comprehension of the study before signing the e-consent form

The study knowledge review questionnaire includes 6 questions supporting the understanding of key study concepts and was developed by Duke’s Program for Empirical Bioethics through iterative cognitive interviews with patients. This process is in keeping with a recent guidance document issued by the FDA for IRBs, in which investigators are encouraged to include “optional questions at any time during the [electronic informed consent] discussion that can be used to help educate the subject about the information presented, as well as assess the subject’s understanding of the informed consent materials” [18]. A screenshot of the online knowledge review questionnaire used in ADAPTABLE is provided in Fig. 2. Assessment of consent was not meant to be a test of participant’s comprehension to be considered before they were randomized. Rather, it was one of the three methods used to enforce understanding. Patients were presented with the question; then, they were provided the correct information and an explanation whether or not they had the correct answer.

The use of e-consent is important for a multi-site pragmatic trial like ADAPTABLE for numerous reasons. Previous studies suggest that conducting electronic informed consent online results in similar or increased comprehension of study concepts in comparison with traditional informed consent [19–21]. In addition, in the multi-center PALM study, video-based consent led to increased representativeness of groups with traditionally lower participation in randomized trials (i.e., non-White and elderly), and sites that approved the video-based consent enrolled their first participants faster [22]. In ADAPTABLE, the e-consent platform additionally aimed to create efficiencies by allowing participants to enroll from anywhere, at the time of their choice, simultaneously reducing study staff’s time allocated to the consent process. Comprehension questions represent a key step to facilitate the IRB acceptance of e-consent, provided they are truly adequate to assess comprehension.

However, many of the participating institutions’ IRBs had limited experience, and no internal protocol in place to address the concept of e-consent. Experience with e-consent imparted a comfort level at some sites, but the idea of patients consenting without immediate access to a member of the study team raised concerns in others. As a result, the e-consent, mainly in the context of remote enrollment, was viewed as an IRB challenge during ADAPTABLE start-up for some participating sites, and local study teams invested substantial time and energy communicating with their IRBs about the nuances of this aspect of the trial.

To pre-emptively address the expected challenges related to IRB evaluation of the e-consent process, the study coordinating center distributed the consent document and a corresponding questionnaire to participating sites and requested that local IRBs review before submission. IRBs expressed key concerns with using a universal version of the consent form. To address this, the steering committee agreed that sites could implement changes to the consent document template allowing locally required language, approved by local IRBs. Finally, some IRBs expressed concern that an e-consent process would introduce selection bias by unintentionally excluding patients who meet all study criteria, but do not use, or comfortably use, the internet [23]. To address this, the steering committee permitted two, rather than one, avenues for enrollment—one for “internet” patients and one for “non-internet” patients. “Internet” participants enroll on their own directly via the study portal. For “non-internet” patients, site research staff facilitate enrollment in the portal and subsequent follow-up visits are conducted through a centralized call center.

To address concerns with the e-consent process, the study coordinating center developed and distributed a separate guidance document about e-consent to be included optionally in IRB submissions. This document outlined key information for IRBs to consider during their review, such as (1) the web-based system’s compliance with federal legislation (e.g., 21 CFR § 11) and nonbinding federal recommendations from the OHRP and the FDA, (2) explanation of supplemental documents that the IRB should review (e.g., video script, storyboards, system mockups), (3) benefits of using electronic informed consent in clinical research, and (4) security, data access, and technical support for the web-based enrollment platform. In future trials involving e-consent, the level of site experience and comfort of IRBs with e-consent needs to be evaluated as early as possible in the process of the protocol development, and their input on the ICF can help setting up a plan to address local, state, and federal regulations.

### IRB and involvement of patient partners

ADAPTABLE is among the growing number of studies that include patients and other community stakeholders as part of the research team [24, 25]. While this strategy has become normal practice in some countries, it remains a fairly new process in the USA. Patient partners can play critical roles in the research process, including articulating research questions; informing research design with patient priorities; improving recruitment and retention strategies; communicating with research participants for purposes of recruitment, consenting, and retention; interpreting data and results from the patient perspective; and disseminating research findings to appropriate community audiences [26, 27]. One patient partner per network (“Adaptors”) was identified with support from the Health eHeart Alliance to help guide the study design process and to ensure that the patient’s voice remained at the center of the research effort [19, 24].

The inclusion of Adaptors as members of the local study team presented administrative challenges within the highly structured regulatory requirements of a study conducted within both academic and non-academic hospital environments. These challenges occurred on one main front: meeting the research and ethical training standards as defined by the IRB (and designed for professionals) for patient partners who may or may not have a medical or clinical research background. As key study personnel, patient partners were subject to the same regulatory requirements and trainings for human subjects’ protections as academic researchers. The online Collaborative Institutional Training Initiative (CITI) program commonly used for annual IRB certification of academic researchers can be burdensome for patient partners who lack research training and experience. For instance, the CITI training includes unfamiliar terminology, requires a significant time commitment for background reading, contains content that could potentially be irrelevant to a patient partner’s role on the project (e.g., research challenges that they are unlikely to encounter), and due to the online format, offers no opportunity for discussion. The Meharry-Vanderbilt Community Engaged Research Core (CERC) and the Vanderbilt University Human Research Protection Program created an alternative to the online CITI training that can be used by patient-partners—a face-to-face version which provides human subjects protection training geared specifically to community member in terms of content, language, time commitment, and opportunity for questions and discussion (Table 2). This resource is particularly relevant for studies in which patient partners interact directly with potential participants.

**Table 2:** Research ethics for community partners: alternative to CITI human subjects protection training

∙ Content vetted and approved by IRB ∙ Taught by project principal investigator ∙ In-person or web conference delivery (for multi-site studies) ∙ Can be tailored with study examples ∙ 50-min presentation (not including Q/A, discussion) ∙ Trainees must pass 14-question quiz with 80% ∙ Trainee assigned institutional credentials for limited system access ∙ IRB notified, trainee added as key study personnel ∙ Annual training options include repeat of community partner module or CITI Community Research module	

*CITI* Collaborative Institutional Training Initiative, *IRB* institutional review board

While many IRBs will accept this training as a substitute for standard courses, local implementation is not immediate and needs to be planned. Incorporating courses similar to Vanderbilt’s training for community members into educational material for patient-partners would help facilitate the distribution of awareness regarding their availability. The improved delivery of initial and recurring ethics trainings for community members involved in research leadership teams could help encourage and simplify the inclusion of patient perspectives in future trials engaging patients among the leadership team.

### Risk determination

Risk determination is an essential step in the IRB process to consider the welfare and safety of participants. Given that aspirin is available over the counter, and that the study evaluates a comparative effectiveness question on two widely available doses, ADAPTABLE presents a unique opportunity to evaluate the consistency and process for risk determination. Namely, the benefits or risks of aspirin are known but the differential benefits or risks of two different doses of aspirin are unknown [28]. There is widespread variation in practice of the use of two different doses of aspirin in secondary prevention of atherosclerotic cardiovascular disease, and community equipoise may be indicated. Randomization, itself, may not necessarily pose additional risk in this setting of an over the counter medication commonly used for patients with coronary disease.

Nevertheless, IRB representatives and study leaders from local sites voiced concerns regarding the risk of the study procedure or randomization of two different over the counter doses of aspirin during the protocol development phase. Some IRBs determined that the study posed greater than minimal risk to patients because the inclusion criteria did not require the patient to be taking aspirin at the time of enrollment, even though it was clinically indicated. In addition, some viewed that individual clinicians may know precisely what is the appropriate dosing for an individual patient despite the lack of supporting evidence. As a result, some sites chose to modify inclusion criteria to require current aspirin use at time of recruitment, and others added safety language to the consent form for aspirin-naive patients to explain risk of bleeding. In these cases, participating sites submitted local protocol addendums explaining the minor modifications they would apply locally to the inclusion and exclusion criteria.

The study coordinating center was committed to resolving the majority of concerns expressed by reviewing IRBs related to risk determination before finalizing the study protocol. However, the steering committee could not resolve all concerns from the participating IRBs within the universal protocol without further delaying study start-up. As a result, risk determination was identified as an IRB challenge during study start-up by local PIs and study coordinators at many sites. Outstanding IRB concerns that were not fully resolved during the study design phase were resolved with one-on-one discussions with local IRBs to submit local protocol addendums explaining such modifications.

### Lessons learned for future trials

Based on ADAPTABLE’s experience, key lessons can be learned to streamline the IRB processes in pragmatic future trials. Without a true reliance model of a central IRB for evaluation and approval of all steps, there are significant challenges meeting the goal of a simple, consistent, and timely review process. Given the current state of affairs of most institutions requiring some local review, it will be important for local IRBs to review the protocol and processes before its finalization for studies incorporating novel methods.

Engaging IRB representatives upfront in the pre-submission process may be helpful. Convening calls before the protocol is submitted at each institution can help understanding individual concerns and gaining traction before review and triage of IRB applications. Sites that adopted this strategy mentioned it minimized issues during the review process by allowing IRB representatives to understand better the more innovative study processes at the time of actual review, and it also helped investigators obtain feedback to optimize their documents before submitting. Conducting education sessions with both local study leadership and IRB staff could also contribute to facilitate the understanding of the innovative methods, their safety, and their acceptability by regulatory bodies and funding agencies (FDA, OHRP, NIH) and thus to minimize the back-and-forth communications with the coordinating center after study submission. Use of a network-wide central IRB with reliance agreements for each site instead of piecemeal solutions across CRNs is the ultimate aspirational goal to streamline the IRB process in future multicenter pragmatic trials, a strategy that is also supported by the federal agencies.

## Conclusion

For pragmatic RCTs incorporating innovative study components, education and attention to potential regulatory or ethical concerns proactively is necessary. True reliance agreements will require cultural changes at the local level to allow another institution to be fully responsible for the review, approval, and oversight of a clinical trial. The experience of ADAPTABLE encapsulates how pragmatic protocol components intended to facilitate the study conduct have been tempered by unexpected and heterogeneous, yet justified concerns raised by local IRBs. Development of effective and engaging communication channels, both formal and informal, between IRB personnel at different sites, local study personnel, and the coordinating center to address issues as early as at the time of protocol design allows to reduce issues with IRB approval. Study teams should work with IRBs to rigorously evaluate the risks/benefits of these studies so that IRBs in the future will have some evidence to make their decisions. As a demonstration project of PCORnet, ADAPTABLE aimed to internalize and operationalize innovative protocol elements to evaluate their feasibility, and to identify key areas for improvement. Integrations of the lessons learned in ADAPTABLE regarding the IRB process for centralized IRBs, informed consent, patient engagement, and risk determination can be emulated and will be instrumental in future pragmatic studies, within or outside the PCORnet network.

## Supplementary Information

**Additional file 1.**

## Data Availability

Data sharing is not applicable to this article as no datasets were generated or analyzed during the current study.

## Abbreviations

CITI: Collaborative Institutional Training Initiative
CTTI: Clinical Trials Transformation Initiative
CRN: Clinical research network
EHR: Electronic health record
FDA: Food & Drug Administration
HPRN: Health plans research networks
ICF: Informed consent form
IRB: Institutional review board
NIH: National Institutes of Health
OHRP: Office of Human Research Protections
PPRN: Patient-powered research network
RCT: Randomized controlled trial
