# Supplementary material for: Streamlining the institutional review board process in pragmatic randomized clinical trials: challenges and lessons learned from the Aspirin Dosing: A Patient-centric Trial Assessing Benefits and Long-Term Effectiveness (ADAPTABLE) trial
Source: Trials. 2021 Jan 25;22:90. doi: 10.1186/s13063-021-05026-w (PMC7831187; doi:10.1186/s13063-021-05026-w)
Supplement: Supplementary file 1 — Additional file 1. [file 13063_2021_5026_MOESM1_ESM.pdf]

APPENDIX B: ADAPTABLE CONSENT FORM, REGULATIONS MARKED

We are asking you to join a research study called ADAPTABLE. The information below explains the study so you can decide if you want to take part or not. Please read it carefully and take all the time you need to decide. Feel free to talk it over with your family, friends, and doctor. If there is anything you do not understand, be sure to ask questions.

Comment [LMB1]: 46.116(a)(1): Study involves research

Comment [LMB2]: 46.116(a)(8): Voluntary

Comment [LMB3]: 46.116 General Regs: Sufficient opportunity to consider whether or not to participate, minimize coercion/undue influence

WHY IS THIS STUDY BEING DONE?

For more than 40 years, doctors have been telling patients with heart disease to take aspirin. For these patients, taking aspirin every day can lower the risk of heart attacks and strokes.

Millions of Americans who have heart disease already take either regular-strength (325 mg) or low-dose (81 mg) aspirin. Many studies have shown that both doses work and both are generally safe. The most common side effect of aspirin is an upset stomach. Aspirin can make you bleed more easily. In rare cases (about 5 in 1,000 people), it can cause dangerous bleeding in the stomach, brain, or other places.

Even though both doses of aspirin are widely used, no one knows which is <u>best</u>. Regular aspirin has a higher risk of bleeding than low-dose aspirin. But no one knows if low-dose aspirin is both safer and works just as well as regular aspirin to prevent heart and blood vessel problems.

The goal of ADAPTABLE is to try to find out which dose of aspirin is best for patients who have heart disease. Patients who join this study will take either low-dose or regular aspirin every day. That way, we can learn which is better in terms of heart attacks, strokes, bleeding, and death.

We expect 20,000 patients with heart disease from across the U.S. will take part in ADAPTABLE.

Comment [LMB4]: 46.116(a)(1): Purpose of research

164.508(c)(1)(iv): Purpose of the requested use/disclosure

Comment [LMB5]: 46.116(b)(6): Approx. number of subjects involved

WHO IS DOING THIS STUDY?

The Patient-Centered Outcomes Research Institute funded this study. The Duke Clinical Research Institute (DCRI) is leading the study, which will be carried out by the Patient-Centered Clinical Research Network (PCORnet). The study directors at Duke are Dr. Adrian Hernandez and Dr. Matthew Roe.

Comment [LMB6]: 164.508(c)(1)(iii): To whom covered entity may make requested use/disclosure

WHAT WILL YOU ASK ME TO DO?

Comment [LMB7]: 46.116(a)(1): Procedures to be followed

If you agree to join ADAPTABLE, here is what will happen:

1. <u>We will ask a few questions to make sure you are a good fit for this study</u>. You cannot take part in this study if you have had problems taking aspirin in the past, take blood thinner medicines, or are pregnant or nursing. You should use birth-control during the study to avoid getting pregnant. If you do get pregnant, let us know right away.

2. We will ask a few things about you and your health. We will ask things like your birth date, sex, and race. We will also ask about other medicines you take, and how you are getting along in daily life. For example, we will ask how you would rate your health; if you are able to do activities like run errands, shop, and take part in social events; and if you feel depressed, tired, or in pain.

   We will ask for your contact information so that we can keep in touch during the study. We will also ask for contact information for a family member in case we cannot reach you.

3. The computer will assign you to take either regular aspirin or low-dose aspirin. Neither you nor your doctor will choose which dose of aspirin you will take. Rather, the computer will assign one or the other randomly. This means every patient has a fair and equal chance of getting either dose of aspirin.

   The reason to use chance, rather than choice, to assign the dose of aspirin you will take is because no one knows which is best for patients like you. Assigning patients randomly helps make sure that the group that takes each dose is about the same. That way, at the end of the study, we can be pretty sure that any differences in health are because of the dose of aspirin—not because the groups were different from the start.

   It is important for the study that you take the dose of aspirin the computer assigns.

4. We will ask you to fill out some short surveys. Once a month, we will ask about any major things that have happened with your health. Every 4 months, we will ask about how you are getting along in daily life. Once a year, we will ask what medicines you are taking. This will happen for as long as you are in the study. You can fill them out on the Web or by phone.

5. We will get some information from other places. Taking part in ADAPTABLE does not require any special study visits or trips to your doctor. But to be sure we get a complete picture of your health:

   - We will get information from your medical record. Examples include information about your health problems, health care visits, medical procedures, and lab results.
   - We will ask for your Social Security number and health insurance ID numbers. We will use these to check other sources (such as health insurance claims) for information about your health.

   We will get these kinds of information from time to time for as long as you are in the study.

WHAT WILL YOU DO WITH MY INFORMATION?

We will store all the information collected for ADAPTABLE in a database at DCRI. Your name and other information that directly identifies you will not be in the database (see "What About My Privacy?"). Researchers will study the information from all the patients who take part to learn more about aspirin, heart disease, and human health.

Comment [LMB8]: 164.508(c)(1)(i): Information to be used/disclosed

Comment [LMB9]: 164.508(c)(1)(i): Information to be used/disclosed

Comment [LMB10]: 164.508(c)(1)(i): Information to be used/disclosed

164.508(c)(1)(ii): Entity(s) authorized to make requested use/disclosure

Comment [LMB11]: 164.508(c)(1)(iii): To whom covered entity may make requested use/disclosure

HOW LONG WILL I BE IN THE STUDY?

We will collect information from you and your records for about 3 years. There is no limit on the length of time we will store your information. We will keep letting researchers use it to learn more about heart disease and human health, unless you ask us to stop.

WHAT ARE THE RISKS?

Even though doctors do not know which dose of aspirin is best, they agree that between 75-325 mg daily is a good idea for most patients with heart disease. The two doses we will compare—low-dose (81 mg) and regular-strength (325 mg) aspirin—are both widely recommended by doctors today.

There are no extra risks from taking aspirin as part of this study compared to taking aspirin as part of your usual care. The main differences are:

- In this study, the computer will assign which dose of aspirin you will take every day
- If you already take aspirin as part of your usual care, you might be assigned to take a different dose (higher or lower) than the one you take now

There will be no other changes to your medical care based on being in the study. If you have side effects or other concerns during the study, you and your doctor are free to decide that you should take a different dose of aspirin or stop taking it altogether.

If you have any questions or concerns about taking aspirin, be sure to talk to your doctor.

WHAT ABOUT MY PRIVACY?

There is a risk that someone could get access to study information we have stored about you, and maybe misuse it. We think the chance of this is very small, but we cannot make guarantees. Your privacy is very important to us. Here are just a few of the steps we will take to protect it:

- We will have your name so we can reach you about the surveys. But when we put all the study information into the database, we will remove your name and other identifiers. We will replace these with a code number. There will be a master list linking the code numbers to names, but we will keep it separate and secure.
- We will store study information on computers with many layers of protection. We will limit and keep track of who sees the information to make sure it is safe.
- Researchers who study information from the database will not know who you are. The information they get will only have the code number, not your name.

Officials from Duke University, PCORnet, or the federal government may review study records to make sure we are doing things the right way. A reviewer who looks at your study record may also need to look at your medical record. Once your information is shared outside the ADAPTABLE team, it may no longer be protected by patient privacy rules (called 'HIPAA'). However, it will still be protected by other privacy rules and agreements.

Comment [LMB12]: 46.116(a)(1): Duration of participation

Comment [LMB13]: 164.508(c)(1)(v): Expiration date for use/disclosure

Comment [LMB14]: 46.116(a)(2): Risks/discomforts

Comment [LMB15]: 46.116(a)(1): Identification of experimental procedures (none)

Comment [LMB16]: 46.116(a)(5) Extent to which confidentiality will be maintained

Comment [LMB17]: 164.508(c)(1)(iii): To whom covered entity may make requested use/disclosure

Comment [LMB18]: 164.508(c)(2)(iii) Re-disclosure no longer protected

ARE THERE ANY BENEFITS?

You will not get direct benefit from taking part in this study. The main reason you may want to join is to help researchers learn about the best dose of aspirin for people with heart disease. The results might benefit patients like you in the future.

You can get general news and updates about ADAPTABLE, as well as helpful information about managing heart disease at [URL].

ARE THERE ANY COSTS OR PAYMENTS?

You will not be paid to take part in this study. If you decide to join, we will ask you to take aspirin every day. We will not provide or pay for this aspirin. However, aspirin is low cost and you can get it at any drugstore or grocery store. You do not need a prescription.

WHAT IF I GET INJURED?

In spite of all safety measures, you might have a reaction or injury from taking aspirin. If such problems occur, you should seek help right away from your usual doctor or place you get care. You or your health insurance will need to pay for this.

WHAT IF I CHANGE MY MIND?

Taking part in ADAPTABLE is your choice. You can choose to join or not. No matter what you decide, now or in the future, it will not affect your ability to get medical care.

If you agree to join ADAPTABLE, you can change your mind at any time. We will tell you if we learn anything new that might make you change your mind about being in the study. If you change your mind, you must let us know in writing. You can email us at [email] or send a letter to [address].

Unless you let us know you want to stop taking part, you will still be in this study even if:

WHO CAN ANSWER MY QUESTIONS?

If you have questions about ADAPTABLE, or any research-related injuries, concerns or complaints, please contact the study directors, Dr. Hernandez or Dr. Roe, at [phone]. You can also contact the ADAPTABLE study office at [phone] or send us a secure message through the ADAPTABLE web site at [URL].

If you have questions about your rights as a research participant, or any research-related concerns or complaints, you can call [IRB contact info].

Comment [LMB19]: 46.116(a)(3): Benefits to participant/others

Comment [LMB20]: 46.116(a)(6): Compensation

Comment [LMB21]: 46.116(b)(3): Additional costs as result of participation

Comment [LMB22]: 46.116(a)(6): Compensation/treatment for injury *[only needed if research involves >minimal risk]*

Comment [LMB23]: 46.116(a)(8): Voluntary

Comment [LMB24]: 46.116(a)(4): Alternatives

Comment [LMB25]: 46.116(a)(8): No penalty/loss of benefits

164.508(c)(2)(ii): Inability to condition treatment, etc. on consent

Comment [LMB26]: 46.116(a)(8): Free to discontinue participation

Comment [LMB27]: 46.116(b)(9):Significant new findings provided

Comment [LMB28]: 46.116(b)(4): Procedure for withdrawal

164.508(c)(2)(i): Right to revoke in writing

Comment [LMB29]: 46.116(a)(7): Whom to contact re: study; whom to contact in event of injury

Comment [LMB30]: 46.116(a)(7): Whom to contact re: subjects' rights
